# Plasticity of network dynamics as observed experimentally requires heterogeneity of the network connectivity pattern

**DOI:** 10.1186/1471-2202-14-S1-P360

**Published:** 2013-07-08

**Authors:** Marco A Huertas, Marshall Hussain Shuler, Harel Z Shouval

**Affiliations:** 1 Department of Neurobiology and Anatomy, University of Texas Medical School, Houston TX, 77030, USA; 2 Department of Neuroscience, The Johns Hopkins University, Baltimore MD, 21205, USA

## 

**Figure 1:**
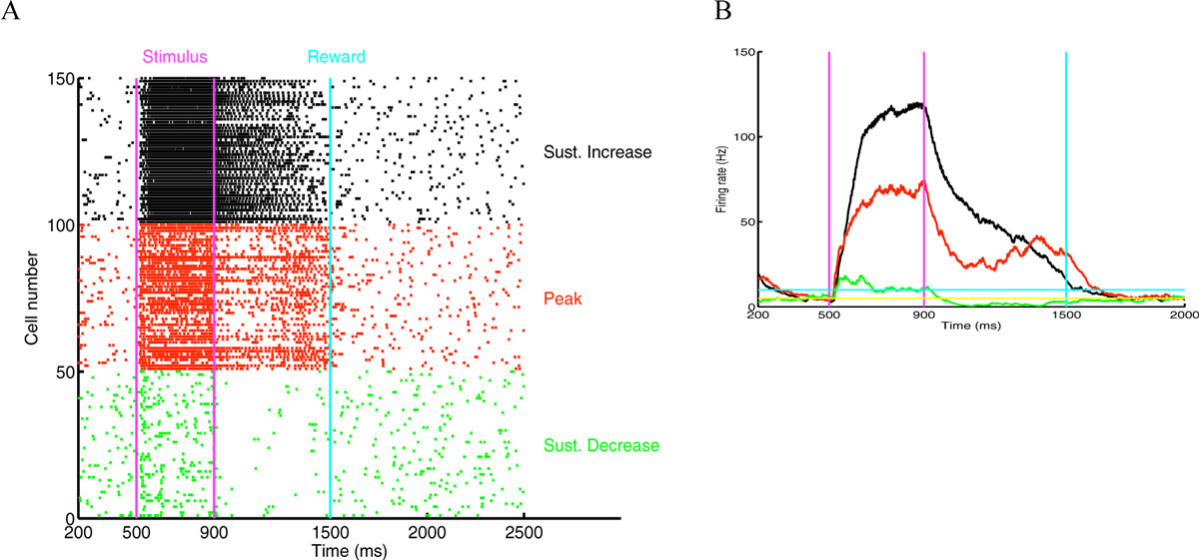
**A. Responses of excitatory population in network model that can report the time of reward (cyan line): SI (black), P (red) and SD (green)**. The network is initially driven by 400 ms of simulated input from LGN corresponding to full-field stimulation (magenta lines) and background noise. **B**. Population firing rate.

Recent in vivo experiments in rats [1,2] have shown that when visual stimulation is paired with a reward signal, neurons in the visual cortex (V1) can exhibit population responses that correlate well with the expected time of the reward. This indicates that V1 neurons have the ability to learn to report the expected times in the seconds range. Data from extracellular recordings suggest that there are at least three different types of responses: 1) a sustained increase (SI) in population firing rate that slowly decays to base level at the time of reward, 2) a sustained decrease (SD) in firing rate until the time of reward and 3) a population firing rate that initially decays and later peaks (P) at the time of reward [1,2]. In a previous computational approach [3,4] we proposed a model that accounts for the mechanism responsible for the SI type of response that allowed the synaptic weights of the lateral connectivity between neurons in an excitatory population to learn the correct values over several trials such that the population firing-rate decayed slowly (due to reverberations) until the time of reward. Here we present an expanded network model that can account for the three types of responses observed in the in vivo experiments (Figure 1). The model consists of two populations of integrate-and-fire neurons, one excitatory and the other inhibitory. We use our previously proposed plasticity rule [4] to learn the excitatory efficacies in the model while keeping the inhibitory connections fixed. We also assume a sparse and heterogeneous connectivity pattern. Under these assumptions the three response types (SI, SD, P) emerge naturally from the learning rule applied to the synaptic weight. This model also exhibits heterogeneity within the different response types, as observed experimentally. The model also exhibits significant robustness to variations of static synaptic weights to and from the inhibitory population and to the presence of noise that generates realistic spike statistics.
